# Smartphone-assisted training with education for patients with hip and/or knee osteoarthritis (SmArt-E): study protocol for a multicentre pragmatic randomized controlled trial

**DOI:** 10.1186/s12891-023-06255-7

**Published:** 2023-03-23

**Authors:** Franziska Weber, Carsten Müller, Carolin Bahns, Christian Kopkow, Francesca Färber, Paul Gellert, Ina Otte, Horst Christian Vollmar, Werner Brannath, Freya Diederich, Stephan Kloep, Heinz Rothgang, Valerie Dieter, Inga Krauß, Corelien Kloek, Cindy Veenhof, Sandra Collisi, Ute Repschläger, Hannes Böbinger, Christian Grüneberg, Christian Thiel, Dirk Peschke

**Affiliations:** 1 Division of Physiotherapy, Department of Applied Health Sciences, Hochschule für Gesundheit (University of Applied Health Sciences), Gesundheitscampus 6-8, 44801 Bochum, Germany; 2 Department of Rehabilitation, Physiotherapy Science & Sports, UMC Utrecht Brain Center, University Medical Center Utrecht, Utrecht University, Utrecht, The Netherlands; 3 Department of Therapy Science I, Brandenburg University of Technology Cottbus-Senftenberg, Senftenberg, Germany; 4 Institute of Medical Sociology and Rehabilitation Science, Charité-Universitätsmedizin Berlin, Berlin, Germany; 5 Institute of General Practice and Family Medicine, Ruhr University Bochum, Bochum, Germany; 6 Competence Center for Clinical Trials Bremen, University of Bremen, Bremen, Germany; 7 Department for Health, Long-Term Care and Pensions, SOCIUM Research Center on Inequality and Social Policy, University of Bremen, Bremen, Germany; 8 Department of Sports Medicine, University Hospital, Medical Clinic, Interfaculty Research Institute for Sports and Physical Activity, Tuebingen, Germany; 9 Research Group Innovation of Human Movement Care, HU University of Applied Sciences, Utrecht, The Netherlands; 10 Referat Projektmanagement und Digitalisierung, Bundesverband selbstständiger Physiotherapeuten – IFK e. V., Bochum, Germany; 11 Innovationsfonds & Produktportfolio, Techniker Krankenkasse, Hamburg, Germany

**Keywords:** Telerehabilitation, Osteoarthritis, Physical therapy modalities, Exercise therapy, Education, Combined modality therapy

## Abstract

**Introduction:**

Hip and knee osteoarthritis are associated with functional limitations, pain and restrictions in quality of life and the ability to work. Furthermore, with growing prevalence, osteoarthritis is increasingly causing (in)direct costs. Guidelines recommend exercise therapy and education as primary treatment strategies. Available options for treatment based on physical activity promotion and lifestyle change are often insufficiently provided and used. In addition, the quality of current exercise programmes often does not meet the changing care needs of older people with comorbidities and exercise adherence is a challenge beyond personal physiotherapy. The main objective of this study is to investigate the short- and long-term (cost-)effectiveness of the SmArt-E programme in people with hip and/or knee osteoarthritis in terms of pain and physical functioning compared to usual care.

**Methods:**

This study is designed as a multicentre randomized controlled trial with a target sample size of 330 patients. The intervention is based on the e-Exercise intervention from the Netherlands, consists of a training and education programme and is conducted as a blended care intervention over 12 months. We use an app to support independent training and the development of self-management skills. The primary and secondary hypotheses are that participants in the SmArt-E intervention will have less pain (numerical rating scale) and better physical functioning (Hip Disability and Osteoarthritis Outcome Score, Knee Injury and Osteoarthritis Outcome Score) compared to participants in the usual care group after 12 and 3 months. Other secondary outcomes are based on domains of the Osteoarthritis Research Society International (OARSI). The study will be accompanied by a process evaluation.

**Discussion:**

After a positive evaluation, SmArt-E can be offered in usual care, flexibly addressing different care situations. The desired sustainability and the support of the participants’ behavioural change are initiated via the app through audio-visual contact with their physiotherapists. Furthermore, the app supports the repetition and consolidation of learned training and educational content. For people with osteoarthritis, the new form of care with proven effectiveness can lead to a reduction in underuse and misuse of care as well as contribute to a reduction in (in)direct costs.

**Trial registration:**

German Clinical Trials Register, DRKS00028477. Registered on August 10, 2022.

**Supplementary Information:**

The online version contains supplementary material available at 10.1186/s12891-023-06255-7.

## Introduction

Osteoarthritis (OA) is a major public health concern, affecting about 12–22% of the population worldwide and causing enormous direct and indirect costs on healthcare systems [1–3]. The prevalence of OA continues to increase due to expanded life expectancy and modifiable risk factors, including insufficient physical activity (PA) and poor diet, all of which promote obesity [4]. OA most frequently occurs in the hip and knee joints and is associated with functional limitations, pain, and restrictions on participation, quality of life and ability to work [1, 5]. Moreover, patients are frequently affected from comorbidities such as hypertension, dyslipidaemia and back pain [6]. National and international guidelines recommend conservative non-pharmacological treatments; especially exercise therapy, education, and increased self-management as first-line treatments for patients with hip and knee OA [7–9]. According to guidelines, individualization of treatment, consideration of patient preferences and facilitated access to ongoing (exercise) programmes should be considered [10]. Exercise therapy and education are safe and effective for patients with hip and knee OA, including those with several comorbidities (e.g., cardiovascular diseases, frailty, and depression) [7, 8, 11, 12]. Furthermore, meeting the World Health Organization (WHO) guidelines on PA, comprising 150 minutes of moderate PA and at least 2 days of muscle strengthening activity weekly is also strongly recommended for patients with chronic diseases such as OA [13].

However, a systematic review and meta-analysis showed that the pass rate (i.e., recommendation or referral) for exercise, education and/or self-management was below 40% for patients with OA [14]. In Germany, 63% of patients diagnosed with hip and/or knee OA received pain medication and only about 36–43% were prescribed physiotherapy [5, 15]. Whether exercise therapy was provided at an appropriate intensity during these physiotherapy sessions, or whether adequate exercises were performed, is unknown. De Rooij et al. (2014) reported that exercise programmes often inadequately address the changing needs of older people or patients with comorbidities, resulting in access barriers, low adherence rates and poor effectiveness [16]. Moreover, a recent representative survey from Germany revealed that not even half of the patients (46%) with OA meets current PA guidelines [17]. Regular, ongoing exercise is strongly recommended for the management of OA [18], but patient adherence to exercise programmes and physiotherapeutic training recommendations tend to decline over time [19, 20]. Non-adherence to long-term exercise regimes may be one reason for only short-term effects of exercise interventions on clinical outcomes in hip and knee OA [21–23]. A systematic review [24] using the Behaviour Change Taxonomy V1, which was initially described by Michie et al. [25], identified behaviour change techniques being particularly effective in physiotherapy interventions to promote PA initiation and maintenance in lower limb OA patients. Techniques associated with interventions being more effective were self-monitoring of behaviour, non-specific reward, and social support (unspecified).

Mobile health (mHealth), including smartphone health applications, web-based health promotion interventions, or virtual reality, can also be used to support health behaviour change and healthcare for people with OA [26]. mHealth is useful for PA promotion and has positive effects on PA levels in people with OA [27]. In physiotherapy care, various stand-alone mHealth interventions exist for the management of OA (e.g., J*oin2Move* [28]) and *My Dear Knee* [29]). Those digital applications provide constant availability of exercises and information, which can increase patient adherence. In addition, the use of mHealth in daily life might complement or even replace parts of personal therapy and increase its effectiveness and efficiency [26]. However, the lack of supervision by trained healthcare personnel, which evidently decreases the quality of movement execution, constitutes a major drawback [30]. Blended care, defined as the combination of digital intervention and in-person treatment, overcomes the lack in quality of movement execution [31]. Blended care in physiotherapy is effective in reducing pain and increasing physical functioning in patients with OA [32]. However, evidence on cost-effectiveness is scarce [32]. In Germany, the guideline on remedies regulates outpatient physiotherapy, but does not include any blended care concepts yet. Therefore, the evaluation of blended care concepts in terms of (cost-) effectiveness and their integration into daily care routine are of great interest from health services research perspective [33–35].

To our knowledge, neither did a previous trial evaluate a blended care concept focusing on education and training in physiotherapy for patients with hip or knee OA in Germany, nor did a previous study examine behaviour change techniques over a 12-month period to sustainably modify and reinforce healthy behaviour. Thus, the SmArt-E trial (“*Smartphone-assistiertes Training mit Edukation*”/ “*smartphone-assisted physical training with education*”), attempts to close these research gaps. The aim of this multicentre, pragmatic, randomized controlled trial (RCT) is to evaluate a digitally assisted therapeutic exercise and education programme compared to usual care in patients with hip and/or knee OA in a real-world physiotherapeutic setting.

The SmArt-E trial is set up according to the Medical Research Council (MRC) framework for evaluating complex interventions and serves three objectives. The first aim is to evaluate the effectiveness of a blended care intervention in patients with hip and/or knee OA compared to usual care on health-related measures with physical functioning and pain at 12-month follow-up being defined as primary outcomes. As a second objective, we assess the cost-effectiveness and cost-utility of the intervention compared to usual care. Thirdly, using a mixed-methods design, a process evaluation of the blended care approach will reveal challenges and barriers related to its implementation that might affect the outcomes.

Primarily, it is hypothesized that the SmArt-E intervention would improve physical functioning and decrease pain more than usual care among patients with hip and/or knee OA. Moreover, we assume that the SmArt-E intervention will further increase quality of life, participation and PA, enhance empowerment, self-efficacy and coping skills, reduce risk factors for joint replacement surgery and retain work ability to a greater extent compared to usual care (secondary hypothesis).

## Methods

### Study design

The study is a three-centre, pragmatic, randomized (1:1), and controlled parallel-group trial with a 12-months intervention period in a real-world physiotherapeutic setting in Germany. The target group (*N* = 330 participants) will be recruited from the public, supported by a statutory health insurance company and by publicity campaigns around the three study centres (see Table 1). Furthermore, general practitioners, family doctors, orthopaedists and physiotherapists will support it. Study centres are located in the German federal states of (1) North Rhine-Westphalia: University of Applied Health Sciences, Bochum (Bochum, Germany: target sample size *n* = 165), (2) Baden-Wuerttemberg: University Hospital, Medical Clinic, Department of Sports Medicine, Tuebingen (Tuebingen, Germany: *n* = 83), and (3) Brandenburg: Brandenburg University of Technology Cottbus-Senftenberg (Cottbus-Senftenberg, Germany: *n* = 82). The SPIRIT (Standard Protocol Items: Recommendations for Interventional Trials) guideline is used for reporting (see Additional file 1) [36]. The manuscript will follow the CONSORT (Consolidated Standards for Reporting Trials) guidelines for transparent reporting of parallel-group randomized trials [37].

**Table 1:** Trial registration data

Data category	Information
Primary registry and trial identifying number	German Clinical Trials Register DRKS00028477
Date of registration in primary registry	10.08.2022
Secondary identifying numbers	NA
Source(s) of monetary or material support	Innovation funds G-BA 10,596 Berlin Germany
Primary sponsor	Innovation funds G-BA 10,596 Berlin Germany
Secondary sponsor(s)	NA
Contact for public queries	Hochschule für Gesundheit Bochum, Department für Angewandte Gesundheitswissenschaften Dr. Carsten Müller Gesundheitscampus 6–8 44,801 Bochum Germany
Contact for scientific queries	Hochschule für Gesundheit Bochum, Department für Angewandte Gesundheitswissenschaften Prof. Dr. Dirk Peschke Gesundheitscampus 6–8 44,801 Bochum Germany
Public title	Smartphone-assisted osteoarthritis training with education (SmArt-E)
Scientific title	Smartphone-assisted training with education for patients with hip and/or knee osteoarthritis (SmArt-E): A multicentre pragmatic randomized controlled trial
Countries of recruitment	Germany
Health condition(s) or problem(s) studied	ICD10: M15 - Polyarthrosis ICD10: M16 - Coxarthrosis [arthrosis of hip] ICD10: M17 - Gonarthrosis [arthrosis of knee]
Intervention(s)	Arm 1: Intervention group: Implementation of the SmArt-E programme. Patients undergo a digitally guided training and education programme. The intervention period is 12 months and consists of the following four components: #1 Physical training (variant A: individual treatment, variant B: group treatment) Neuromuscular training (NEMEX program) comprising trunk stability, lower extremity strengthening, postural control, functional training. This is an evidence-based training programme whose feasibility and effectiveness has been demonstrated in previous international studies. #2 Education (variant A: individual treatment, variant B: group treatment) Education/ seminars on topics related to pain physiology, pain management, exercise and sport in osteoarthritis, lifestyle adaptation, empowerment/ self-efficacy, weight management. #1 & #2 = > Variant A: takes place during the initial phase of the SmArt-E intervention and comprises 6 to 8 appointments with a duration of 40 minutes each in individual mode, preferably 2 units per week. Variant B: takes place during the initial phase of the SmArt-E intervention for 6 weeks and comprises 2 sessions per week with a duration of 80 minutes each in a group setting. #3 Smartphone-assisted support (app). Takes place over the entire intervention period. Training: digital follow-up of the above-mentioned exercises, available in video, image and text form. Participants are regularly reminded of the exercises (via reminder function). They can give feedback (e.g. on exercise performance, satisfaction, complaints). Physiotherapists thus gain insight into the progress and status of their patients and can make appropriate training adjustments (progression/ regression). Education in text and video form on topics such as “What is osteoarthritis?”, “Exercise with osteoarthritis”, “Origin and function of pain”, “Healthy body weight and other health benefits”... Physical activity: Via the app, participants can select a preferred physical activity that they would like to perform or increase. This is supported on the one hand by the educational content and physiotherapeutic care, and on the other hand by activity tracking and the app’s reminder function. Video coaching (chat option with a physiotherapist), recording and documentation, user evaluation (feedback) of the program, and individual components. Refresher (takes place after 6 months, up to three appointments of 40 minutes each): selected content from training management, motivational support, self-management, evaluation of current progress, review of goal achievement, support for continued independent progress. #4 Individual physiotherapy on-demand component: only for participants of the group treatment.
	Arm 2: control group: usual care
Key inclusion and exclusion criteria	Sex: All Minimum Age: 38 Years Maximum Age: no maximum age Additional Inclusion Criteria: Medically/physician-diagnosed osteoarthritis (ICD-10-GM: M.15 polyarthrosis, M16. - coxarthrosis, and M17. gonarthrosis) and fulfillment of the American College of Rheumatology criteria for knee/hip osteoarthritis. Age > 50 and > 38 years for hip and knee osteoarthritis, respectively.
	Exclusion of participants who have already had joint replacement of the affected index joint (total joint arthroplasty).
Study type	Interventional
Date of first enrolment	Nov, 2022
Target sample size	330
Recruitment status	Recruiting ongoing
Primary outcome(s)	#1a: Hip Disability and Osteoarthritis Outcome Score (HOOS) OR #1b: Knee Injury Osteoarthritis Outcome Score (KOOS) AND #2: Numeric Rating Scale (NRS) to assess pain severity
Key secondary outcomes	Three assessments are scheduled: 1. T0 assessment = following enrolment and before randomization/ start of the intervention; 2. T1 assessment = 3 months after the start of the intervention/ after completion of the in-person intervention; 3. T2 = after completion of the 12-months study period. 1. HOOS or KOOS and NRS after 3 months 2. Physical performance tests (modified Y-Balance Test; 30-second Sit-to-Stand Test) after 3 and 12 months 3. Daily physical activities (assessment is device-based and questionnaire-based) after 3 and 12 months Questionnaire surveys: 4. Action and coping planning (Action Planning & Coping Planning) at 3 and 12 months. 5. Quality of life/ health status (European Quality of Life 5 Dimensions 5 Level Version (EQ-5D-5L), Patient Global Assessment (PGA) and self-rated change in health status after 3 and 12 months 6. Assessment of coping strategies for pain and its evaluation (Pain Catastrophizing Scale, PCS) after 3 and 12 months 7. Self-report behavioural automaticity index (SRBAI) after 3 and 12 months 8. Assessment of the general improvement of the disease (Patient Acceptable Symptom Scale, PASS) after 3 and 12 months 9. Self-efficacy/ empowerment (Arthritis Self-Efficacy Scale, ASES) after 3 and 12 months 10. Patient satisfaction (ZUF-8) and an additional item assessing the patient’s perspective on the satisfaction with the results of the treatment after 3 and 12 months 11. Physical activity health competence (short form) after 12 months 12. Usability: System Usability Scale (SUS) after 3 months and mHealth App Usability Questionnaire (MAUQ) after 12 months 13. Questions on health care expenditures (financial expenditures due to osteoarthritis-related knee/hip problems for utilization of health care providers, therapeutic treatments, aids, etc.) for the period 12 months before study inclusion and during the 12-months intervention period

### Study apparatus

#### Intervention

##### Summary of the intervention

The three core components of the SmArt-E intervention comprise a neuromuscular exercise programme (MVI.PG.ZZ Assisting and leading exercise for sensations related to muscle and movement functions) combined with disease specific education (SH1.PM.ZZ Education about mobility, unspecified) and promotion of daily PA (graded or non-graded PA) (VEB.PM.ZZ Education to influence physical activity behaviours), conveyed through blended care using a smartphone app.

**Fig. 1:**
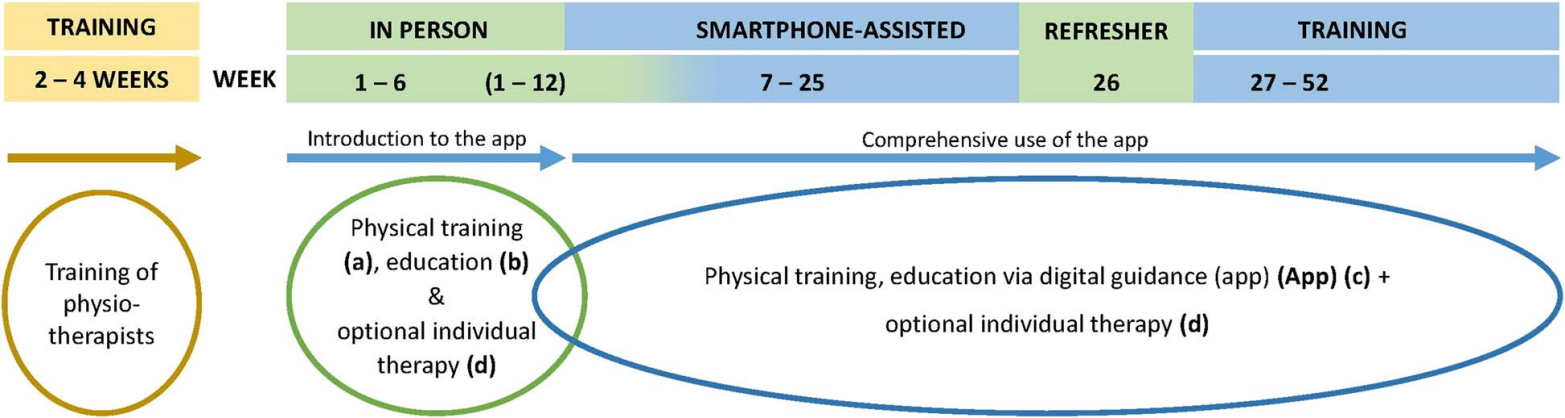
Flow of the SmArt-E intervention programme

The one-year intervention is divided into two phases (1) in-person sessions with a physiotherapist on a regular base and (2) a subsequent training and education phase, which is essentially assisted by the app (see Fig. 1). All elements being assisted by the app are initially introduced in the in-person physiotherapy sessions. The in-person treatment comprises 80-minute group-sessions twice a week for 6 weeks, or 4-8 sessions of 40 minutes within 12 weeks on an individual base, respectively. Participants are asked to do additional exercises at home to accumulate three training sessions per week. Moreover, they should choose a preferred activity to increase or maintain their habitual PA level. Upon completing personal treatment, patients exclusively use the app during the smartphone-assisted training phase at home. At 6 months, 1–3 individual refresher sessions are being held to increase commitment and adherence throughout the blended care phase. This is accomplished by providing feedback, promoting self-efficacy by highlighting accomplishments and barriers overcome, reviewing and adjusting goals, as well as discussing and supporting problem solving.

##### Content of the intervention

The intervention is based on the e-Exercise intervention from the Netherlands, but further adapted and extended, and consists of a training and education programme [32]. In detail, it comprises a progressive exercise design, based on the *neuromuscular exercise* (NEMEX) programme [38], with increasing intensity levels over the 12-months period, either a graded or non-graded PA programme, as well as ongoing OA-related educational content, provided in five sequential protocols. Each exercise set consist of three blocks, which include (1) warm up, (2) main exercises and (3) cool down exercises. The second block integrates two strengthening exercises (hip and knee strengthening), a core stability exercise, a functional exercise (e.g. standing up from a chair) or a balance exercise. Those exercises can be increased or decreased, for instance for the knee extension exercise, it is possible to use an elastic band to increase the intensity of the exercise. The training parameters are set by default and are increasing from time to time. However, supervising physiotherapists can adapt the protocols as needed. The intervention features ongoing support for patients by their supervising physiotherapists to answer questions, adjust exercises, or discuss problems and barriers. Further details on the individual components of SmArt-E are shown in Tables 2 and 3. The role of the physiotherapy practices is to carry out the intervention including the subsequent digital support.

**Table 2:** Detailed prescription of the components of the SmArt-E intervention

Intervention: 52 weeks ***of SmArt-E*** training and education	Number/ Dose:	Period:	in total:
**Physical training (option A: individual therapy, option B: group therapy)** Neuromuscular training (NEMEX program) from the areas (trunk stability, strengthening of the lower extremities, postural control, functional training). This is an evidence-based training programme, the feasibility and effectiveness of which has been proven in earlier international studies [18].	**A: **4–8 sessions of 20 minutes each **B:** 12 sessions of 40 minutes each	**A:** 12 weeks **B:** 6 weeks	**A:** 4–8 sessions of 20 minutes each **B:** 12 sessions of 40 minutes each
**Education (Variant A: individual therapy, variant B: group therapy)** Education/seminars on topics of pain physiology, dealing with pain, exercise and sport in osteoarthritis, lifestyle adjustment, empowerment/ self-efficacy, weight management	**A:** 4–8 × 20 min **B:** 12 × 40 min	**A:** 12 weeks **B:** 6 weeks	**A:** 4–8 × 20 min **B:** 12 × 40 min
**Smartphone-assisted support (app)** **Training:** digital continuation of the above-mentioned exercises available in video, image, and text formats. The participants are regularly reminded of the exercises (reminder function). Participants can provide feedback (e.g. regarding performing exercises, satisfaction, pain and exertion). In this way, physiotherapists can acquire an insight into the progress and status of their patients and can make training adjustments (progression/regression) as necessary. **Education** in text and video formats on topics such as “What is osteoarthritis?”, “Exercise with osteoarthritis”, “The origin and function of pain”, “Healthy body weight and other health benefits”... **Physical activity:** Participants can use the app to select a preferred physical activity that they would like to do or augment. This is supported on the one hand by the educational content and physiotherapeutic support, and on the other by activity tracking and the app’s reminder function. **Video coaching** (possibility to chat with and speak to a physiotherapist), **Recording and documentation, user evaluation (feedback)** of the program and individual components	Self-directed in part by the participants. Recommendations for training and physical activity are conveyed. Physical training 3x/ week; physical activity 3x/week.	6/12 weeks (getting to know the app and putting trust with it) 46/40 weeks (excluding training, education, and physical activity with this component)	See column 2
**Refreshers** Selected content from training control, motivational support, self-management, evaluation of current progress, verification of goal achievement, and support for ongoing independent continuation.	1–2 × 40 min (depending on need)	2–3 weeks after 6 months	
**Individual physiotherapy** Demand component: for participants of both (group and individual variant)	Dosage and number as is the case with usual care	depending on needs	depending on needs

**Table 3:** Description of the SmArt-E intervention using the TIDieR checklist

Item	Description
1. Brief name	Smartphone-assisted osteoarthritis training with education (SmArt-E)
2. Why	Exercise therapy and education have been shown to be effective for patients with hip and knee osteoarthritis. However, current programmes often fail as no sustainable lifestyle changes can be achieved. The use of mobile health (mHealth) has the potential to support self-management and the process of behavior change in patients with chronic diseases beyond the traditional borders of physiotherapy practice. The blended care approach combines in-person and digital interventions and can increase acceptance and adherence to exercise programmes.
3. What: Materials	The SmArt-E intervention includes three core components: (a) physical training, (b) education and (c) smartphone-assisted support via a mobile phone app. The (a) physical training is based on the NEMEX program and includes neuromuscular exercises for trunk stability, lower limb strengthening, postural control and functional training. The (b) educational component comprises topics such as pain physiology, lifestyle adjustment, empowerment/self-efficacy, and weight management. The intervention is supported by (c) a mobile phone app that includes a home-based exercise programme and education in text and video formats. The app also aims to promote physical activity and offers the possibility of video coaching via chatting with a physiotherapist. The following materials are used in the intervention: • Training equipment: resistance bands (3 levels), chair, step-ups with modifiable levels, balance pad, weights. • Presentation materials: Posters and presentations, to be used in the in-person phase for teaching educational content. • Mobile phone app “HealthTrain”: The app will be available for download on any mobile phone. It supports the digital continuations of the NEMEX exercises, which are available in video, image, and text formats. Educational content is delivered in text and video formats on topics such as “What is osteoarthritis?” and “Exercise with osteoarthritis”. Activity tracking and the app’s reminder will support the participants to increase their physical activity. • Participants’ manual: A manual containing general information about the study (e.g., aims, timeline), safety instructions and a guide on how to use the mobile phone app. • Physiotherapists’ manual: A manual containing information about the study (e.g., aims, timeline) and safety instructions. There is also a summary of the educational content and all exercises including pictures and written instructions. In case participants do not want to use their private mobile phone or do not have a suitable device, they will be provided with a mobile phone for the duration of the study. The physiotherapy practices are equipped with a tablet on which the participating physiotherapists can use the “HealthTrain” app.
4. What: Procedures	The SmArt-E intervention is divided into an in-person phase and a self-training phase. If more than four persons are interested in participating at the same time, the intervention can take place in a group setting, otherwise it is conducted in individual physiotherapy sessions. Each session consists of an educational and exercise part. The first session is also about getting to know the app and its functions. During the self-training phase, the participants continue the intervention with the support of the mobile phone app. In case of questions or problems, it is possible to contact the physiotherapist via the chat function of the app. Six months after the start of the intervention, refresher sessions will take place to repeat specific educational content. If necessary, additional individual physiotherapy can be delivered.
5. Who provided	Trained physiotherapists perform the in-person exercise programme and education as well as the refresher sessions. Physiotherapists receive 2 days (4 hours each) of training specific to the SmArt-E intervention from skilled members of the study team. Individual therapy (if required) can be delivered by any physiotherapists without special training.
6. How	During the in-person phase, the intervention is provided in either individual (one-to-one) or group setting (4–8 participants). After the in-person phase, SmArt-E also includes the phase of self-training planned from the distance with the support of a mobile phone app and with remote physiotherapeutic support using the app’s chat feature.
7. Where	Three study centres: Bochum/Dortmund/Essen (North Rhine-Westphalia, Germany), Cottbus/Senftenberg (Brandenburg, Germany), Tübingen/Reutlingen (Baden-Württemberg, Germany) The in-person sessions are delivered in an outpatient physiotherapy setting. During the self-training phase, participants decide themselves where to exercise, probably in their own homes. Individual physiotherapy and refresher sessions will also take place in the respective physiotherapy practice.
8. When and how much	The SmArt-E intervention is scheduled to last 52 weeks. In-person phase (6–12 weeks): Individual therapy: 4–8 sessions of 40 min each for 12 weeks (20 min exercise, 20 min education) Group therapy: 2 sessions of 80 min each per week for 6 weeks (40 min exercise, 40 min education) Self-training phase (52 weeks): Self-directed in part by the participants. Desired training frequency: physical training 3x/week for 30–40 min; physical activity 3x/week. Refresher: 1–2 sessions for 40 min, 6 months after intervention start
9. Tailoring	During the in-person phase, the physiotherapists personalize the intensity of the exercises to each participant’s capacity. Parallel to the in-person sessions, participants start exercising at home with the support of the app. For the first 4 weeks, the focus is on form and technique rather than on effort. Subsequently, the exercise programme will change every126 weeks, adjusting the exercises themselves, the number of repetitions and sets. The physiotherapists are able to choose one out of two sets of exercise with different intensities. The participants are also instructed to self-tailor the exercise programme themselves by choosing a level of intensity within each exercise (e.g. using a resistance band with higher/lower resistance, increasing/decreasing the range of motion) provided by the app. The education content is standardized.
10. Modifications	N/A
11. How well: Planned	To ensure standardized delivery of the SmArt-E intervention, training for physiotherapists follows a standardized protocol and will be delivered by the same project members. All physiotherapists get a handbook that summarizes all exercises and education material. The smartphone-assisted support via the app plays a key role in improving participants’ adherence to the SmArt-E intervention. To encourage the participants to stay active, continue their exercises and reinforce the educational content, the app is designed to remain engaging over the 52-week period: There is a changing exercise programme every 12 weeks and new educational videos or texts at least every 2 weeks. The app also integrates various motivational and volitional techniques of behavior change to increase exercise fidelity (e.g., goal setting, action planning, self-monitoring of behavior). Moreover, the refresher sessions aim to support for ongoing independent continuation of the programme. Participants‘training and app adherence will be assessed using training diaries and user data from the app.
12. How well: Actual	N/A

##### SmArt-E app

**Fig. 2:**
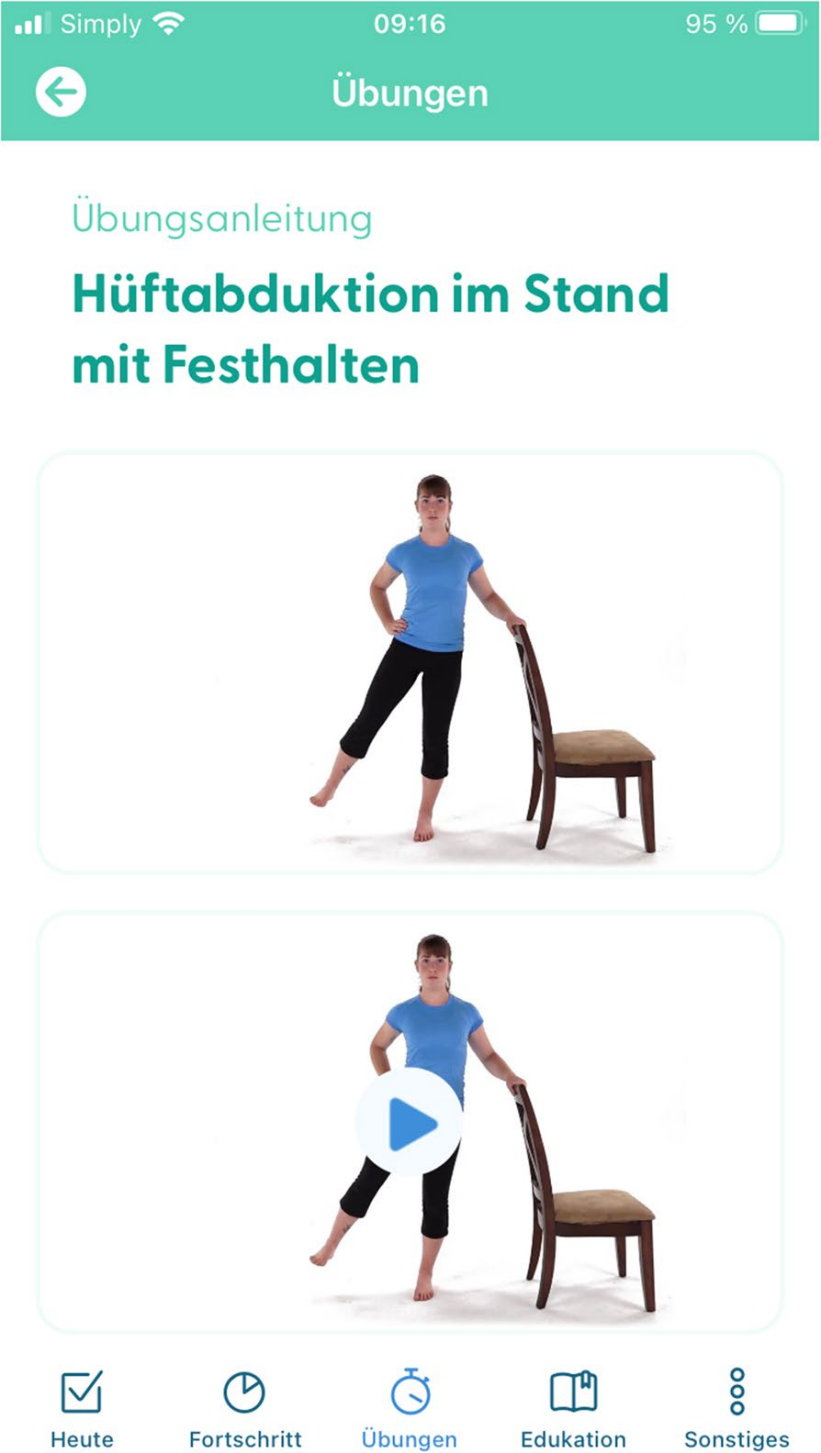
Screenshot of the SmArt-E app showing the exercise feature

**Fig. 3:**
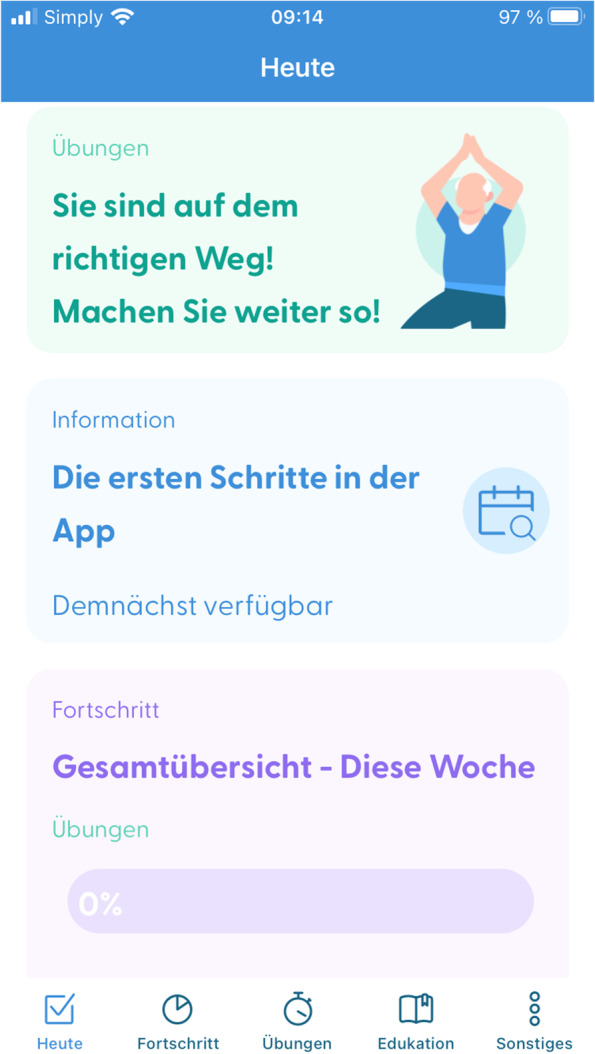
Screenshot of the SmArt-E app showing the today view

As early as the in-person treatment starts, patients shall also use the app for regular exercises, PA, and education at home. The app additionally holds a chat and videoconference feature to connect patients and their physiotherapists on demand. Moreover, the app features support behaviour change through weekly exercise and PA goals, scheduling of exercise, self-monitoring of one’s performance and automated feedback on progress including positive reinforcement through compliments for goal achievement. Figures 2 and 3 show the today view and the exercise feature of this app as of December 2022, respectively.

##### Behaviour change techniques

Physiotherapists are introduced to strengthen participants’ self-efficacy regarding independent training and PA. To support both phases, the initiation as well as the maintenance of regular exercise and PA by patients, behaviour change techniques are incorporated into the intervention as part of the in-person physiotherapy sessions as well as the subsequent smartphone-assisted training phase. Based on effective behaviour change techniques [24], physiotherapists guide participants to set long-term personal goals, to make action plans for exercises and PA (i.e., action planning) and to establish them as habitual routines by implementing them according to personal preferences and easily compatible with daily life. This planning is based on providing basic information on OA, pain and chronic disease management. Furthermore, possible barriers and problem-solving strategies to deal with them in the sense of coping planning are discussed.

#### Training of physiotherapists to provide the SmArt-E intervention

A structured training builds on existing knowledge and competences to qualify physiotherapists in the SmArt-E intervention. Therefore, an 8-hour training (including pre- and post-processing) in groups of up to ten physiotherapists on the study itself, the components of the intervention, the behaviour change techniques in use and an app introduction will be conducted. This will be followed by a 1.5-hour refresher course after 3 months after the intervention start to expand knowledge on techniques for behaviour and lifestyle changes. Learning objectives for the initial training are to become familiar with the (1) exercise programme and educational content of the SmArt-E intervention, (2) possibilities to adapt, supervise and individualize the exercise programme and daily PA, (3) features of the app, (4) blended care approach, and (5) self-management strategies in SmArt-E. Moreover, physiotherapists should be able to (6) independently adapt the preselected exercises of the app to the individual needs and preferences of the patients, and (7) to empower participants to perform physical exercises on their own, assisted by the app. Furthermore, they should be trained (8) to support participants to cope with their disease independently and (9) to perform health-enhancing PAs regularly (graded or non-graded PA programme). The learning objectives of the refresher course encompass (1) additional motivational strategies, (2) specific coping strategies to enable participants to integrate self-managed exercises into their daily life with adequate intensity. Furthermore, physiotherapists should (3) support participants remotely in terms of exercise programme design and educational content. Additionally, they shall (4) support participants in behavioural and lifestyle changes, (5) promote PA, and (6) enable participants in the retention of behaviour change and development of coping skills in the long term, e.g., strategies to resolve emerging problems.

#### App adaptation and enhancement: thinking-aloud method

The study app is being further developed based on an existing Dutch app (e-Exercise) in close collaboration between the research team and the technical partner *HealthTrain* (Utrecht, Netherlands). Once the app is ready for implementation (Android and iOS), we use the thinking-aloud procedure to assess its usability. Therefore, five randomly selected participants are to accomplish selected tasks within the app while expressing their thoughts aloud. The feedback is recorded with the help of audio recordings and evaluated in terms of qualitative content analysis. This sample size is sufficient to log 85% of the usability problems [39].

### Usual care

The control group receives usual care, which might comprise physiotherapy sessions, but also other treatments like injections, acupuncture, or no treatment at all. An additional 45-minute consultation with a physiotherapist on PA-recommendations for OA including informational material, access to the app, and a smartphone if sought, is offered following the final assessment.

### Outcomes (measures)

Table 4 shows descriptive variables, as well as primary and secondary outcomes. Assessors receive 2 h of training on all aspects of the assessment to ensure a high level of standardization.

**Table 4:** Overview of outcome measures, screening instruments and descriptive measures over the course of the study

		PS	TS	T_**0**_	T_**1**_	T_**2**_
	*Sociodemographic and health-related data*					
	Date of birth, age, and sex			X		
	School-leaving qualification; academic grades; employment; living conditions (i.e., living alone/ in a nursing home); monthly net income;			X		
	German-speaking		X			
	*Medical and medication information*					
	Height; weight; body mass index; health status; co-morbidities; walking aids			X		
	OA-related treatment history and surgery; previous joint replacement			X		
	Screening for study eligibility and absence of contraindications	X	X			
	*Physical functioning*					
P	Hip Disability and Osteoarthritis Outcome Score (HOOS)			X	X	X
P	Knee injury and Osteoarthritis Outcome Score (KOOS)			X	X	X
	*Pain and tiredness*					
P	Numeric rating scale (NRS)			X	X	X
	*Physical capacity*					
S	Modified Y-Balance-Test			X	X	X
S	30-Second Chair Stand Test (30s CST)			X	X	X
	*Physical activity*					
S	ActiGraph 3 GT3X tri-axial accelerometers and activity diary			X	X	X
S	Activity questionnaire			X	X	X
	*Action planning and coping planning*			X	X	X
S	*Questionnaire*					
	*Health-related quality of life, health status*					
S	European Quality of Life 5 Dimensions 5 Level Version (EQ-5D-5L)			X	X	X
S	Patient Global Assessment (PGA)			X	X	X
S	Subjective change of health status				X	X
	*Coping strategies*					
S	Pain Catastrophizing Scale (PCS)			X	X	X
	*Psychosocial status*					
S	Self-Report Behavioral Automaticity Index (SRBAI)			X	X	X
S	Patient Acceptable Symptom State (PASS)			X	X	X
S	Arthritis Self-Efficacy Scale (ASES)			X	X	X
	*Satisfaction*					
S	ZUF-8 questionnaire + 1 item for patient perspective on satisfaction with treatment results				X	X
	*Usability*					
	System Usability Scale (SUS)				X	
	mHealth App Usability Questionnaire (MAUQ)					X
	*Health literacy*					
	European Health Literacy Survey (HLS-EU-Q16)			X		
	eHealth Literacy Scale (eHEALS)			X		
S	Movement-related Health Literacy			X		X
	*Technology readiness*					
	Technology commitment			X		
	*Health Economic Evaluation* Questions on out-of-pocket expenses and resource use			X		X
	*Health insurance routine data* Data comprise information on costs, diagnoses, procedures and the scope of services, e.g., number of doctor visits, length of hospital stay.			X		X
	*Interaction between user and intervention*					
	Thinking Aloud-Approach			X		

*Abbreviations*: *PS* pre-screening by general practitioner, *TS* telephone screening, *T*_*0*_ baseline assessment, *T*_*1*_ 3-month assessment, *T*_*2*_ 12-month assessment, *P* primary outcome measure, *S* secondary outcome measure

#### Primary outcomes

The primary outcomes are physical functioning and pain. Physical functioning will be assessed using the subscale “function in daily living” of the Hip Disability and Osteoarthritis Outcome Score (HOOS) [40] or of the Knee injury and Osteoarthritis Outcome Score (KOOS) [41], depending on the index joint. Both questionnaires are validated in German language comprising 40 and 42 items, respectively, distributed across the five subscales pain, symptoms and function in daily living, sports activities and quality of life [40, 41]. Each item is scored on a five-point Likert scale (0 = extreme symptoms/problems; 4 = no symptoms/problems). Hence, lower scores indicate higher impairments. Pain will be assessed using an 11-item numeric rating scale (NRS) [42, 43]. Patients select a number (0–10 integers) that reflects their pain intensity best on a horizontal bar [43], ranging from zero (“no pain”) to ten (“worst pain imaginable”) and related to the previous 24 hours [42, 43].

#### Secondary outcomes

##### Knee−/hip-related health, symptoms, and functionality

The HOOS/KOOS subscales pain, symptoms, sports activities and quality of life of the will be assessed as secondary outcomes of the study [40, 41].

##### Physical performance

The *Lower Quarter Y-Balance Test (YBT)* is a simplified version derived from the Star Excursion Balance Test that represents a valid and reliable assessment of lower extremity dynamic neuromuscular control among patients with OA [44, 45]. The YBT uses a testing kit and a revised protocol to improve reliability and screening speed [45, 46]. Participants perform the test while standing in a single-leg stance and pushing a rectangular box with their foot along the three axes of the kit. Based on three attempts into each direction and on each leg, the greatest reached distance in relation to the individual leg length as well as the according between-limb asymmetry will be assessed to evaluate the intervention effects.

The *30-Second Chair Stand Test* (30-CST) is a valid and reliable lower extremity physical performance measure in which participants stand up and sit down as often as possible within 30 seconds with their arms crossed in front of their chest. The 30-CST demonstrate better responsiveness in detecting physical performance changes compared to other measures and is appropriate for older adults over the age of 60 years [47].

##### Physical activity (PA)

Daily PA will be assessed using ActiGraph GT3X triaxial accelerometers [48], successfully used in previous clinical trials including patients with knee and/or hip OA [49]. Participants wear the accelerometer attached to a waist belt for ten consecutive days throughout their waking hours, except for showering or swimming. Additionally, participants will be asked to complete a short activity diary including wearing time, unusual activities, or activities difficult to detect, as well as reasons and duration for removal of the device. Data will be recorded at a sampling rate of 100 Hz. After returning the accelerometer and diary, data will be downloaded, processed, and analysed, for which the Freedson thresholds will be used. 0–99 counts per minute (CPM) indicate sedentary behaviour, while values between 100 and 1951, 1952–5724, 5725–9498, and values ≥9499 CPM indicate light, moderate, vigorous, and very vigorous PA, respectively [50]. Valid PA assessments require a minimum wear time of at least 7 days, 10 h per day [51].

Additionally, we will use a questionnaire on daily PA focusing on leisure and work, comprising nine items that are part of the “German Health Update” (GEDA) study of the Robert Koch Institute [52], and a single question derived from the Global Physical Activity Questionnaire developed by the WHO on daily sitting time [53].

##### Action & coping planning

Planning can be divided into action planning and coping planning, and can be considered a prospective self-regulatory tool that can help turning goals into behaviour [54]. Two scales measuring action planning and coping planning [54] will be used following an adaptation to planning regular PA, consisting of three items each that are rated on a four-point Likert scale ranging from “completely disagree” [1] to “totally agree” [4]. The action planning scale assesses the extent to which a person has made specific plans about when, where, and how they will be physically active on a regular basis. The coping planning scale assesses the extent to which a person has made specific plans about how they will cope with potential barriers to regular PA.

##### Quality of life/health status

The *European Quality of Life 5 Dimensions 5 Level Version (EQ-5D-5L)* questionnaire is valid and reliable for the assessment of the health status in clinical and health economic studies [55, 56]. The EQ-5D-5L covers five domains (mobility, self-care, usual activities, pain/discomfort, and anxiety/depression). Further, it includes five levels referring to the severity of restrictions, based on which the EQ-5D-5L-index is determined that expresses the health status ranging from zero to one with one indicating the best possible health status. This index will be used to calculate Quality Adjusted Life Years (QALYs) [57].

The *Patient Global Assessment (PGA)* is used to quantitatively assess the general health status using a single question from the EQ-5D-5L health questionnaire in its German version [56, 58, 59]. Patients score their current global health status on a visual analogue scale ranging from 0 to 100 mm using the anchors worst and best possible. The *subjective health status change* will be assessed regarding the change in general, the change in pain, and the change in physical functioning (walking). According to Angst et al. (2017) [60], participants will be asked: “How is your health (pain, physical function) today compared to three months ago regarding OA in your affected knee joint or hip joint?”. Response options include the anchors “much better”, “slightly better”, “about the same”, “slightly worse”, and “much worse” [60].

##### Coping strategies for pain

Pain catastrophizing will be assessed using the *Pain Catastrophizing Scale* (PCS) comprising the three subscales helplessness, magnification, and rumination [61]. In 13 items, participants rate the frequency of pain experiences on a five-point Likert scale ranging from zero (mild symptoms) to four (worst symptoms). Adding the individual items results in a total score ranging from zero to 52. Higher scores indicate more pain catastrophizing characteristics. A reliable and validated German version of the PCS is available [62].

##### Psychosocial status

The *Self-Report Behavioural Automaticity Index* (SRBAI) is a four-item subscale of the Self-Report Habit Index (SRHI) that reliably captures habitual behaviour patterns and can be used to predict future behaviour or to track habit formation or disruption [63, 64]. Being more succinct than the SRHI, the SRBAI is easier to administer, but still sensitive to effects that characterize habits [63].

The *Patient Acceptable Symptom State* (PASS) uses a single question to assess the level of symptoms at which patients consider themselves healthy [65]. The PASS will be assessed using the global question: “Assuming you remain in the present condition for the next few months, do you believe that your current state is acceptable?”. A dichotomous yes/no response option indicates the patient’s satisfaction with their current symptoms state.

The *Arthritis Self-Efficacy Scale* (ASES) was originally developed as a 20-items questionnaire to assess OA-specific self-efficacy in patients and to explain changes resulting from (educational) interventions [66]. A shortened and validated German version comprises eight items rated on a visual analogue scale ranging from one (very uncertain) to ten (very certain), with higher scores indicating increased perceived efficacy [67].

##### Physical activity related health competence

The construct *PA-related health competence* comprises the three domains movement control, control competence, and self-regulation, required to perform health-promoting PA on a regular base [68]. A shortened 14-items version derived from the validated original instrument is available in German language for assessing PA-related health literacy in rehabilitation and prevention settings [69, 70].

##### Patient satisfaction

The *ZUF-8* measures global, unidimensional patient satisfaction with received care [71]. It covers eight items that are rated on a four-point Likert scale ranging from one (least favourable) to four (most positive characteristic), available in German language with excellent psychometric properties [71, 72]. For evaluating the app satisfaction, participants of the intervention group will respond to three additional questions, analogously to the original ZUF-8 items, but related to the app. The questions are: 1. “To what extent did the app meet your needs?”, 2. “Would you recommend the app to a friend if they needed similar help?” and 3. “Would you use the app again if you needed help?”. To add the patient perspective on their satisfaction with the treatment results, the following item will accompany the ZUF-8: “How satisfied are you with the results of your treatment?”, rated on a five-point Likert scale ranging from “very satisfied to very unsatisfied” [73].

#### Covariates

##### Health literacy

Health literacy will be assessed using the *European Health Literacy Survey* (HLS-EU-Q16) [74]. The questionnaire addresses the four dimensions of general health literacy encompassing access, understanding, assessment, and use of health information in disease prevention, health promotion, and healthcare [74]. The participants rate tasks and activities related to healthcare, disease prevention, and health promotion on a four-point Likert scale. The *eHealth Literacy Scale* (eHEALS) is developed to meet the growing requirement to assess digital health literacy [75]. The questionnaire includes eight items and assesses the perception of knowledge and skills in finding, evaluating, and using electronic health information on a five-point Likert scale. eHEALS is available in German language [76] and represents a valid and reliable scale for perceived measurement of eHealth literacy among adult populations and patients with chronic diseases [77].

##### Technology commitment

Technology commitment is thought to predict the successful use of new technologies, particularly at older ages. A 12-item scale for measuring *technology commitment* with good psychometric properties has been developed and validated in German language [78]. The scale addresses three different facets of handling technology, comprising technology acceptance, technology competence beliefs, and technology control beliefs.

### Participants

#### Participant timeline

**Fig. 4:**
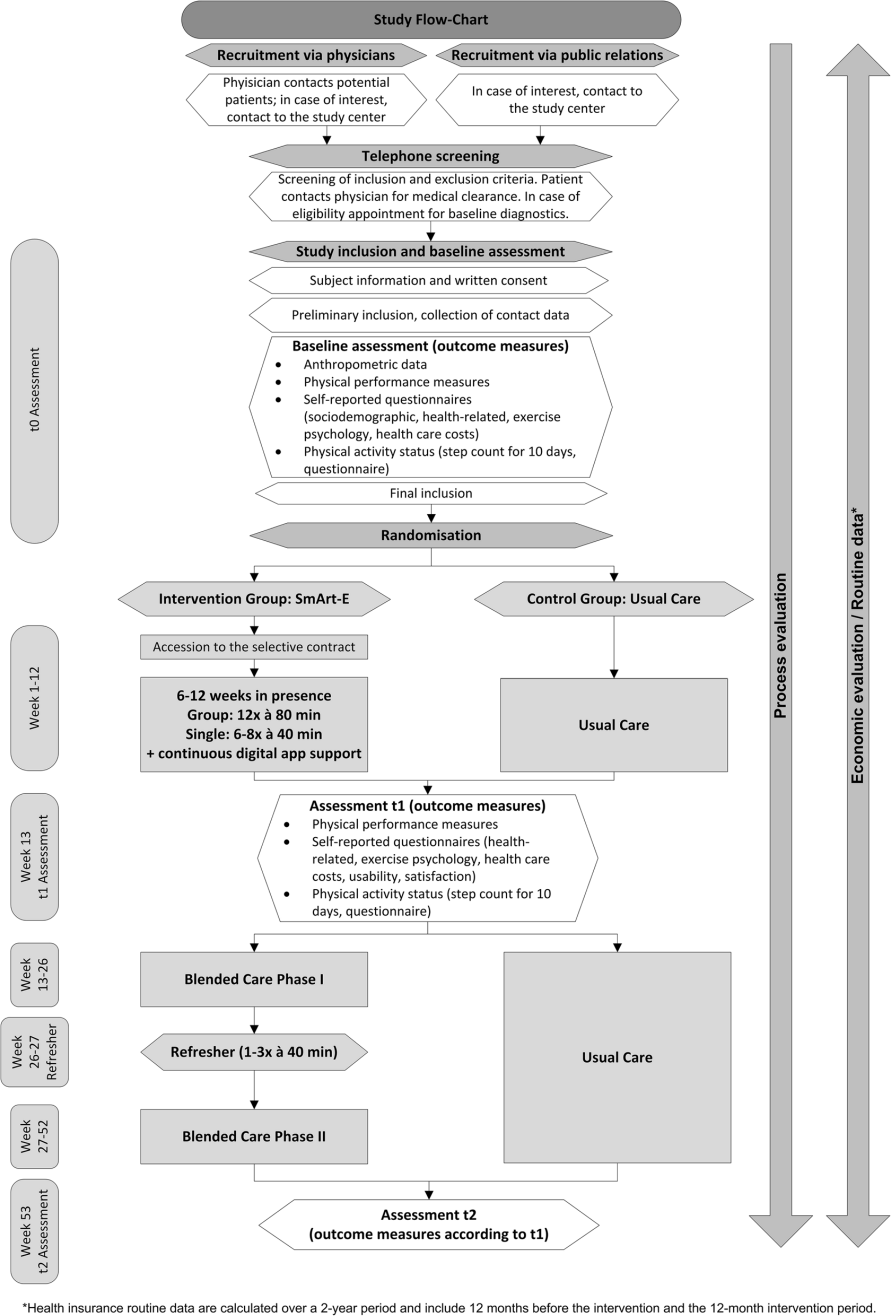
Patient flow of the SmArt-E study

Study participation begins with an initial assessment (t0), followed by randomization of participants. The SmArt-E intervention expands over 12 months with follow-up assessments at 3 (t1) and 12 months (t2) after the start of the intervention and starting with in-person physiotherapy. A refresher session is held after 6 months (see Fig. 4).

#### Sample size

We aim to recruit 330 patients, equally distributed among the intervention and control group. The sample size calculation assumes an effect size of Cohen’s *d* = 0.34 for the comparison of the *SmArt-E* intervention and usual care for the primary outcomes of pain and physical functioning after 12 months. This assumption is derived from a recent meta-analysis involving 44 clinical studies evaluating internationally established, comparable exercise therapy compared to *do-nothing* or *sham* control conditions and a dropout rate of 15%, resulting in effect sizes of *d* = 0.49 for pain and *d* = 0.52 for physical functioning [23]. A comparable RCT with digital support demonstrate similar results, in which the authors find a greater pain reduction of approximately − 1.0 (±2.3) points on a 11-point NRS in the intervention group compared with the control group, corresponding to an effect size of *d* = 0.44 [33]. Regarding physical functioning, an effect of *d* = 0.66 is observed. We assume smaller effect sizes for the SmArt-E intervention as this will be compared to current usual care, which should be more effective than *Do-Nothing* or *Sham*. Based on a *t*-test for independent samples, one-sided *α* = 0.05 and *β* = 0.80, a sample size of *n* = 274 is necessary when considering the number of outcomes and Bonferroni corrections (*n* = 137 per group). Target sample size is increased to *n* = 330 to compensate for dropouts of up to 20%. Additional power is gained by statistically accounting for age, gender, and the baseline value of the respective outcome.

#### Recruitment

The three study centres engage in the recruitment of participants and physiotherapy practices by means of letter mailings, press releases, print media (flyers, posters), information events, newsletter, websites, an animated explanatory film and an article in the customer magazine of the health insurance company (Techniker Krankenkasse (TK)). In addition, a personalized mailing will be sent from the TK to eligible patients in the regions of interest. Cooperating physicians are asked to make their patients aware of the study. Patients interested in participating must contact their trial centre for a comprehensive informed interview addressing questions and study eligibility.

**Fig. 5:**
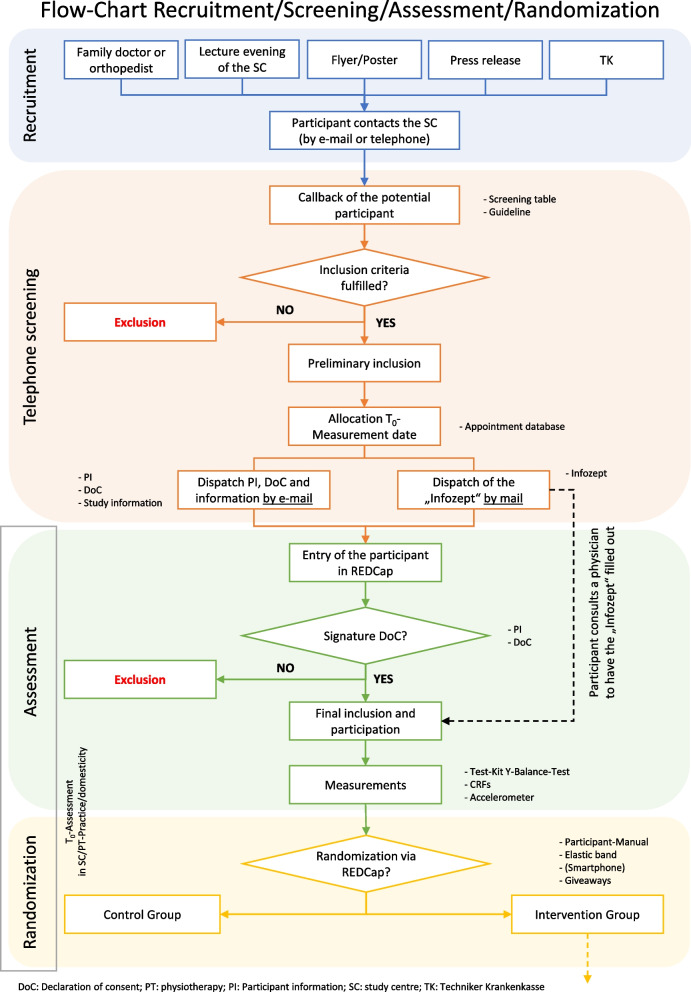
Flow-Chart of the recruitment, screening, assessment and randomization process

Patient recruitment will extend over 9 months (November 2022 to June 2023). An extension may be required, should the COVID-19 pandemic result in restrictions at cooperating institutions that cannot be controlled by the study centres. Figure 5 provides an overview of the entire recruitment process.

#### Eligibility criteria

Patients with OA at the hip and/or knee joint diagnosed by a physician according to the criteria of the “American College of Rheumatology” [79, 80] are eligible for this trial. The minimum age for patients with hip and knee OA is 50 and 38 years, respectively. The responsible physician must verify the patient’s eligibility for inclusion in advance with the “Infozept” (green prescription). Additionally, participants need to be insured with TK, and have sufficient German language skills for the assessments and the intervention of at least B2 level according to the Common European Framework of Reference for Languages [81]. In addition, participants need internet access at home and a SIM card (see Table 5). A smartphone can be provided, if wanted.

**Table 5:** Inclusion and exclusion criteria for study participation

Inclusion criteria	Exclusion criteria
• Physician-diagnosed OA of the hip and/or knee according to the clinical criteria of the American College of Rheumatology (ACR) [79, 80] ICD-10-GM: M15 polyarthrosis, M16 coxarthrosis; M17 gonarthrosis	• Patients with a fixed date for a replacement surgery of the affected joint or already having a total replacement of the affected joint
• Patients with hip OA aged > 50 years; patients with knee OA aged > 38 years according to the ACR [79, 80]	• Patients without internet access at home
• Medically eligible to participate in the SmArt-E intervention	• Inability to understand the German language (B2-level)
	• Patients who are not medically eligible to participate in the Smart-E intervention (e.g. contraindications like cardiovascular or neurological diseases)

#### Randomization and blinding

At t0 (initial assessment) the assessors will allocate participants to the intervention or control group by block randomization using REDCap (Vanderbilt University, Nashville, Tennessee, USA), for which a 1:1 allocation ratio stratified by the affected joint (hip vs. knee), sex, and study centre will be applied. Participants of the intervention group will be treated in the nearest participating physiotherapy practice.

#### Data collection and management

The Competence Centre for Clinical Trials Bremen (KKSB) links data from different sources for the summative, the health economic and the process evaluation (data acquired during baseline and follow-up assessments, routine data from TK, and app-based data). In the assessments, we use machine-readable paper documents, pseudonymized in the Trust Centre at the KKSB and forwarded to the data management department of the KKSB for conducting the summative evaluation following checks for missing data and plausibility to ensure data quality.

#### Data protection

In accordance with data protection legislation, the Trust Centre of the KKSB destroys the key at the earliest possible time when no further requests are open. Data access is exclusively granted to authorized employees of the KKSB involved in this project. All study data is stored in a secured network. A joint controller agreement regulates joint responsibilities among the consortium partners in the processing of personal data pursuant to article 26 General Data Protection Regulation (GDPR).

#### Statistical analysis

The primary outcomes comprising physical functioning (HOOS/KOOS) and pain reduction (NRS) at 12 months from baseline are compared between the intervention and control group using Bonferroni-adjusted analysis of covariance with gender, index joint, allocation (individual/group treatment), baseline values (pain and physical functioning) and the study centre as covariates. In sub-group analyses, corresponding models will be applied only to patients with hip or knee OA and only to women or men, respectively. Wherever required, differences in the effects are tested by expanding the overall model with corresponding interaction terms. Study centre effects are studied in a similar way. A secondary analysis will examine intervention group differences in the time course of pain and physical functioning, including all assessment appointments and using mixed models. Effects on the secondary outcomes are investigated descriptively (and without Bonferroni correction) using appropriate regression models. The analysis is primarily based on the *intention-to-treat* principle. Missing values will be considered by multiple imputation and *maximum likelihood* principle, respectively. If large numbers of imputations will be necessary, a robustness analysis with alternative imputation strategies will be carried out. All statistical analyses are defined in a Statistical Analysis Plan prior to the completion of patient recruitment.

### Health economic evaluation

A cost-effectiveness and a cost-utility analysis will be performed from the societal perspective and from the perspective of the German statutory health insurance. Direct costs (inpatient and outpatient services, rehabilitation, home care, medication, and aids) will be calculated using data from the statutory health insurance and from the patient questionnaire. The questionnaire includes questions on patient out-of-pocket expenses and on home care. Both cost components are relevant from the societal perspective. Home care will be valued according to the opportunity cost approach. Indirect costs will be calculated using data from the statutory health insurance and quantified using the human capital approach. All time components will be valued by average German wages. Costs will be collected at t0 and t2 for the previous 12 months.

In the cost-effectiveness analysis, the effects will be measured using the primary outcomes of physical functioning and subjective pain, that is, the HOOS [40], the KOOS [41], and the NRS [42]. In the cost-utility analysis, the effect will be measured using QALYs [57] based on the EQ-5D-5L [56]. In both analyses, costs and consequences will be compared by calculating the incremental cost-effectiveness ratio. To account for uncertainty, cost-effectiveness acceptability curves will be calculated based on net-benefit regressions and sensitivity analyses will be performed [82, 83].

### Process evaluation

The process evaluation takes place in parallel to the study and serves as a quality criterion, as well as to increase the measurability of the study. The process evaluation examines the mechanisms by which the SmArt-E programme interventions bring about change [84] and how the context affects the implementation and potential outcomes [85]. The process evaluation will evaluate (1) whether the programme is implemented as intended (*fidelity*), (2) to what extent the implementation succeeds (*dose*), (3) whether the targeted addressees are reached (*reach*), and (4) which factors hinder or facilitate the implementation and intervention outcomes (*context*). By identifying context factors needed to be considered in the interpretation of the results, the process evaluation allows concluding on the generalizability and transferability of the intervention [85]. The focus of the process evaluation lies on the three key components of the intervention: The physical training of the patients, their educational training, and their use of the app. A mixed-methods-approach will be used to collect qualitative and quantitative data regarding attitudes and opinions of all target groups towards the intervention components, the communication and interaction among the participants, influential context factors, or perceived barriers during the intervention [86, 87].

The mixed-methods-approach comprises three main methodological components: First, semi-structured guided interviews of 45 to 60 minutes will be performed with 15 patients, 15 physiotherapists, and staff from the trial centres. Secondly, a questionnaire based on the results of the first interviews will be handed out to all patients, and physiotherapists to determine their attitudes, opinions, and the acceptance of the intervention on a quantitative basis. As part of this survey, previously collected data such as socio-demographic factors, quality of life, coping strategies and (digital) health literacy will be referenced to identify potential patient clusters. Thirdly, homogeneous focus group interviews are conducted with groups of physiotherapists and groups of patients separately. The focus group interviews intend to clarify unresolved questions from the interviews and questionnaires, to validate the results from the initial interviews and to reveal information that emerge through the dynamics of a discussion within a peer group. For each of the three study centres, one group of ten physiotherapists and one group of ten patients will be formed leading to a total of six focus group interviews. Each focus group interview will last between 60 and 90 minutes. The quantitative and qualitative data collected in the process evaluation will be complemented by focused ethnographic participatory observations in each study centre at one point in time [88].

#### Usability

As part of the process evaluation, the *System Usability Scale* (SUS) will additionally be used among participants of the intervention group as a well-established self-report instrument to evaluate perceived user-friendliness (usability) [89]. The scale covers ten items, uses a five-point Likert scale with anchors ranging from “strongly disagree” to “strongly agree”, and is available in German language [90]. The *mHealth App Usability Questionnaire* (MAUQ) was developed to assess the perceived usability of digital health applications, comprising 18 items rated on a seven-point Likert scale from one (strongly agree) to seven (strongly disagree) [91]. The questionnaire is not (yet) available in a validated German-language version but will be used in a self-translated version that has already been used in previous studies [92].

## Discussion

This pragmatic multicentre RCT will evaluate the (cost-) effectiveness of a blended care intervention, with in-person and app-assisted therapy components in a real-world physiotherapeutic setting.

The benefit of conservative treatment options for patients with OA has already been proven, however, they are still underutilized. Further, digital care has shown its value in various settings and several countries and proven to be useful to increase the sustainability of interventions [26, 93]. However, exclusively digital care is not appropriate for every patient [28]. In this regard, blended care might render positive outcomes and overcome potential barriers by integrating in-person and digital therapy [32, 94].

### Purpose

The primary goal of the SmArt-E intervention is to reduce pain and improve physical functioning. Additionally, in the long term we expect the intervention to postpone or prevent joint replacement surgeries and retain work ability, as was demonstrated in a previous study combining exercise therapy with education [21, 95]. Compared to exercise therapy alone, the integration of behaviour change techniques and graded PA, core elements of the SmArt-E intervention, decrease the likelihood for joint replacement [21, 95] surgeries. Likewise, a RCT demonstrated that an exercise intervention improved work ability, underlining beneficial effects of physical exercise in patients with OA in retaining work ability [96]. Secondly, many patients with OA also suffer from comorbidities, most often hypertension, dyslipidaemia and back pain [6], for which PA, exercises, and education proved to be beneficial treatments [97]. Thus, we expect SmArt-E to be an effective intervention for patients with OA. We further aim to reveal potential barriers and facilitators to prepare this intervention for implementation in a healthcare setting. For this purpose, the process evaluation shall identify important details to be considered during the roll out and in relation to design and arrangement.

### Expected challenges

Even though this RCT is well designed and planned to determine the effectiveness under the conditions of everyday physiotherapeutic care in Germany, some challenges remain. First, timely recruitment of physiotherapy practices in all three study centres will be crucial regarding the training and education of therapists before the intervention starts. As an incentive for participation, each physiotherapy practice will be equipped with a tablet for the duration of the intervention and receives remunerations comparable to/slightly higher than remunerations according to regular care. Secondly, only patients with hip and/or knee OA insured with TK will be eligible to participate, posing additional challenges with respect to the limited recruitment period of 9 months. However, an initial potential analysis conducted by TK revealed areas with high density of eligible patients. This allows us to focus our recruitment efforts on regions with about 30,000 potential study participants who meet the inclusion criteria.

Other typical challenges might be low adherence or even non-use of the app. To improve adherence to exercise therapy and maintain positive effects on outcomes on the long-term, it is considered important to implement strategies to support lasting behaviour change and integration of exercise and PA into patients’ daily lives [19]. Therefore, behaviour change techniques were included in the intervention that have been applied in PA promoting interventions that resulted in improved adherence in patients with chronic musculoskeletal conditions and specifically OA, according to systematic reviews [24, 98]. Previous studies revealed low adherence rates for exclusively digital care [28]. We aim to overcome this barrier by an initial in-person treatment period, followed by a blended-care intervention, in which patients keep in touch with their therapists, receive exercise reminders, and use behaviour change techniques, including weekly reviews on completed exercises, PA, and educational content, action planning, goal setting, and others. Additionally, we expect the refresher sessions at 6 months to further motivate participants continuing the intervention with increased programme adherence compared to exclusively digital interventions [28].

The process evaluation serves to capture the adherence and further aspects to better understand the factors, which may enhance or mitigate intervention effects.

### Health economic evaluation

An economic evaluation, as it will be conducted in this study, has been recommended as one of the most urgently required strategies to close the gap between evidence and clinical practice [99]. In general, exercise interventions with or without education appear to be cost-effective in patients with hip and knee OA if compared to physician-delivered usual care [100]. The combined supervised patient education and exercise therapy programme GLA:D, a programme similar to NEMEX, has been shown to be cost-effective in the Danish setting [101]. However, blended physiotherapy has not been shown to be cost-effective if compared to usual physiotherapy [102]. The cost-effectiveness of a programme like SmArt-E that combines both approaches is, therefore, not a priori clear. Economic evaluations of OA interventions should use administrative data for collecting health resource use and cost-questionnaires to collect patient out-of-pocket expenses. In addition, sensitivity analyses should be performed [100]. The results of this economic evaluation will provide a meaningful comparison of SmArt-E’s costs and consequences by taking timely recommendations for economic evaluations of OA interventions into account.

### Strengths and limitations

There are several strengths of this study. First, conducted as a pragmatic, multicentre RCT, SmArt-E is more powerful and less prone to several biases (e.g., local effect bias, selection and performance bias, or concomitant therapy bias) compared to single-centre studies. Second, we evaluate an evidence-based exercise intervention [32], incorporating the NEMEX programme [38] and effective behaviour change techniques [103]. Third, given an overall 12-month intervention period, the implementation of refresher sessions after 6 months shall increase programme adherence. Fourth, SmArt-E as novel healthcare will be conducted and evaluated in physiotherapy practices and thus in a real-world environment, supporting its integration into regular healthcare, in case SmArt-E will be evaluated positively. Furthermore, structured feedback in form of exercise diaries, documentation sheets, questionnaires, and process audits from participants (patients, physical therapists, and physicians) will be collected as part of the process evaluation, which can be used for further adaptation, implementation processes and a direct rollout in the healthcare setting. In terms of limitations, even though the study takes place in a real-world setting, physicians and physiotherapists participating in the trial may be not completely representative of all physicians and physiotherapists in Germany. Further, the trial centres will provide implementation support. Findings may therefore not be generalized to other physicians and physiotherapists with limited interest or capacity. As typical of healthcare service research, participants may take part in other healthcare offers, which might delude specific SmArt-E effects. More specifically, control group participants may be sensitized to the relevance of exercise and PA. Obviously, we will ask for participation in any healthcare during the study period. Although follow-up is 12 months, long-term effects of the intervention, such as rates of post-study surgery and disease specific healthcare costs, may not be captured adequately. If the 12-month evaluations are promising, a long-term follow up should be considered. Further limitations include socially desirable reporting at both the level of physiotherapists and patients. Based on our results, as usual, prediction of benefits and harms of the intervention will be possible on a group, but not an individual level.

In brief, this study will provide insight into the (cost-) effectiveness and implementation of a blended care intervention for patients with hip and/or knee OA, which is designed to decrease pain and increase physical functioning. If evaluated positively, the SmArt-E intervention will improve healthcare for patients with OA and alleviate the pressure on healthcare services. Decreasing pain and increasing physical functioning could prevent joint replacement surgeries, retain work ability, improve quality of life and participation, and reduce healthcare costs. Furthermore, as an evaluated smartphone-assisted training and education programme, it can be smoothly implemented into daily physiotherapeutic practice and is therefore ready to use.

#### Trial status

Recruitment of participating physiotherapy practices has ended in August 2022. The official start of participant recruitment was mid of November 2022; enrolment of the last participant is planned for June 30, 2023. By the time of submission (January 10, 2023), *n* = 85 participants have already been enrolled in the trial.

## Supplementary Information

**Additional file 1.**

**Additional file 2.**

## Data Availability

The de-identified datasets generated and/or analysed during the current study may be made available from the corresponding author upon reasonable request once the study has been completed.

## Abbreviations

30-CST: 30-Second Chair Stand Test
ASES: Arthritis Self-Efficacy Scale
CPM: Counts per minute
eHEALS: eHealth Literacy Scale
GDPR: General Data Protection Regulation
HLS-EU-Q16: European Health Literacy Survey
HOOS: Hip Disability and Osteoarthritis Outcome Score
ICER: Incremental cost-effectiveness ratio
KKSB: Competence Centre for Clinical Trials Bremen
KOOS: Knee Injury and Osteoarthritis Outcome Score
MAUQ: mHealth App Usability Questionnaire
mHealth: Mobile health
MRC: Medical Research Council
NEMEX: Neuromuscular Exercise
OA: Osteoarthritis
PA: Physical activity
PASS: Patient Acceptable Symptom State
PCS: Pain Catastrophizing Scale
QALY: Quality Adjusted Life Year
RCT: Randomized controlled trial
SRBAI: Self-Report Behavioural Automaticity Index
SRHI: Self-Report Habit Index
SUS: System Usability Scale
TK: Techniker Krankenkasse
WHO: World Health Organization
YBT: Y-Balance Test
