# Supplementary material for: Smartphone-assisted training with education for patients with hip and/or knee osteoarthritis (SmArt-E): study protocol for a multicentre pragmatic randomized controlled trial
Source: BMC Musculoskelet Disord. 2023 Mar 23;24:221. doi: 10.1186/s12891-023-06255-7 (PMC10034894; doi:10.1186/s12891-023-06255-7)
Supplement: Supplementary file 2 — Additional file 2. [file 12891_2023_6255_MOESM2_ESM.pdf]

HS Gesundheit
BOCHUM

Hochschule für Gesundheit
University of Applied Health Sciences
Gesundheitscampus 6 – 8
44801 Bochum
hs-gesundheit.de

DAG · Department of Applied Health Science

Franziska Weber und Dr. Carsten Müller
Research Associates
SmArt-E Project

Hochschule für Gesundheit
Gesundheitscampus 6 – 8 · 44801 Bochum

T +49 234 77727 - 692
Smart-e@hs-gesundheit.de

Bochum · 12 January 2023

Trial information for participants

SmArt-E: Smartphone-assisted physical training with education

Dear participant,

we would like to invite you to participate in the scientific trial *"SmArt-E"*. You have been diagnosed by a physician with hip and/or knee osteoarthritis. We aim to examine the effectiveness of a training and education program for people with osteoarthritis compared to usual care. The program is digitally accompanied by an smartphone app. We are pursuing three goals:

1. The first goal of *"SmArt-E"* is to increase the physical functioning and activity of the participants, reduce pain, improve quality of life and social participation, and prevent unnecessary surgeries.
2. Furthermore, a health economic review of *"SmArt-E"* should provide information as to whether the program can reduce health expenditure for the treatment of osteoarthritis and thus provide financial relief for the insurance pool.
3. Finally, any difficulties that may arise when using the app that could affect the success of the digital support in this program should be identified.

The study is conducted at the University of Applied Health Sciences in Bochum (HS Gesundheit), funded by the innovation fund of the Federal Joint Committee (Gemeinsamer Bundesausschuss), and led by Prof. Dr. Dirk Peschke, Professor of health services research at HS Gesundheit. Further study centers are located in Tübingen and Cottbus-Senftenberg.

A total of 330 participants will take part at the three study sites. Your participation in the study will not affect your medical treatment, about which you have already been informed by your physician.

By participating in this study, you will be **randomly** assigned to one of two groups, either the intervention group or the control group.

The **intervention group** carries out the *"SmArt-E"* program and has full access to the associated study application (app) via smartphone. The app can be installed on the private smartphone.

Alternatively, a smartphone intended for this purpose is provided free of charge and does not have to be returned after the study is completed.

The **control group** receives usual care as treatment. After completion of the study, the control group receives individual health advice of 45 minutes, full access to the study app and, if required, a smartphone on which the app can be used.

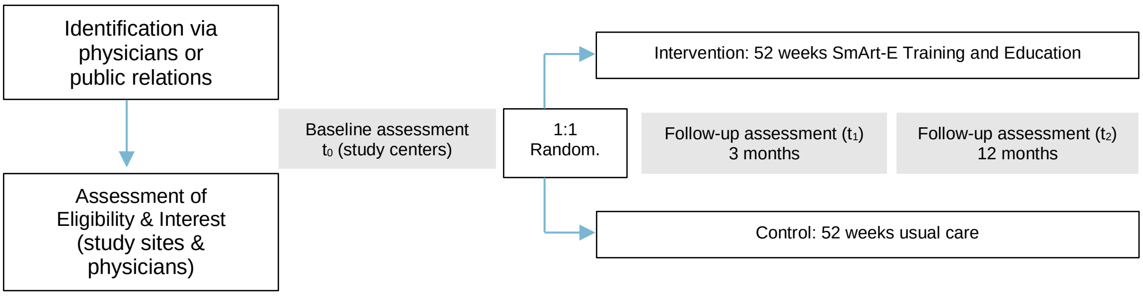

Detailed description of the "SmArt-E" programme

- The *"SmArt-E"* intervention runs for twelve months and consists of four components, of which #1-#3 are integrative parts of each face-to-face session:
- **Component #1:** *Physical training.*
  The physical training takes place in the initial phase (6-12 weeks) as group training with up to six participants or as individual training, with 1-2 appointments per week and is guided by physiotherapists.
- **Component #2:** *Education.*
  This component supports the participants throughout the intervention and takes place in the form of individual or group sessions. Participants receive information on topics such as dealing with pain, exercise for osteoarthritis, and much more.
- **Component #3:** *Digital support.*
  Throughout the intervention, participants have access to the app with controllable training programmes, educational modules and occasional video coaching sessions with their physiotherapists. The app should be used 1-2 times a week.

After the initial phase, the participants carry out the training independently at home with the support of the app. In addition, after six months, a "refresher" of one to three individual sessions of 40 minutes each takes place to support the continuation of the self-training.

- **Component #4:** *Individual physiotherapy.*
  Individual physiotherapy can be used in consultation with the therapist if component #1 is conductedin a group setting.

If you are interested in our study, we ask you to take part in **three separate measurement dates** within the next twelve months: at the beginning (*t_0*), after three months (*t_1*) and at the end of the study after one year (*t_2*). To keep the effort for you low, you can attend the appointments at the study centre, or at a participating physiotherapy practice near you.

Here you will perform performance tests under the guidance of our trained staff and complete questionnaires. For these examinations we plan a duration of 2.5-3 hours in the first appointment. (*t_0*) and up to 1.5 hours for the follow-up appointments (*t_1* und *t_2*).

We would also like to ask you to wear an activity monitor (ActiGraph) to record your everyday activity for ten days after the above-mentioned measurement appointments. It is a small and lightweight monitor that is attached with a strap and and you can put it on and take it off yourself (dimensions: 3.8 x 3.7 1.8 cm, weight: 27 grams). The device measures accelerations ("activity counts"), which are then read out on a PC and translated into sedentary, moderate or strenuous physical activity using an algorithm. This data is only accessible to project staff and is stored using your study ID (pseudonymised).

As part of the study, personal data is collected and evaluated that is necessary to achieve the goals mentioned above. This includes:

- self-reported socio-demographic and health-related data
  (e.g. age, gender, employment, information on health situation)
- Data from the performance tests to describe the interaction of strength, mobility and coordination

Routine data from your health insurance company from the twelve months before and the twelve months during the intervention. These contain information on costs, diagnoses, procedures and the scope of services (e.g. number of doctor visits, length of hospital stay). They cover the following service areas: outpatient medical services, inpatient and outpatient hospital care, rehabilitative measures, home care as well as medicines, remedies and aids. In addition to health economic effects, changes in the use of services due to the intervention should be recognized. Your name and date of birth will also be used to link with other data collected as part of the study. However, this information is only received by a trust agency and not by any person entrusted with the evaluations (Competence Center for Clinical Trials Bremen (KKSB), see data protection). This means that no conclusions can be drawn individually.

In addition, we would like to test the user-friendliness of the app with six people in the Bochum area. The participants will be given ten tasks to carry out in the app (e.g. "Navigate to the start screen") and to comment on (e.g. "What do you think about the clarity of this start page"). The duration of this test is about 15-20 minutes.

This method helps us to improve the user-friendliness of the app. You can revoke your participation in this at any time, even without giving reasons. In this case, you will not suffer any disadvantages and you do not have to withdraw from the study.

Process evaluation

In the course of *"SmArt-E"* you ***may*** be invited to participate in the process evaluation. In this case, we would be pleased if you accept the invitation and share your experiences with the

"SmArt-E" intervention with us. The results of the study should contribute to improved care for patients with osteoarthritis in the future. By voluntarily participating in the process evaluation, you support us in assessing the feasibility of the study.

1. **Individual interviews (45-60 min) with 15 selected study participants from the intervention group**
2. **Questionnaire on the experience with *"SmArt-E"***
3. **One focus group interview (60-90 min) per study site with a maximum of 10 participants per group at the study centre**

You will receive further information on the procedure of the components of the process evaluation, instructions for participation and the informed consent form to be signed in case you are invited to participate in the process evaluation.

Your participation in the study is voluntary.
You will only be included if you give your written consent. If you do not wish to participate in the study or wish to withdraw from it at a later date, you will not suffer any disadvantages. You can withdraw your consent verbally or in writing at any time, even without giving reasons. In this case, we would be pleased, if you were available to us for a final appointment.

The study project was submitted to the responsible ethics committee. It did not raise any objections.

Possible risks, complaints and side effects

The *"SmArt-E"* intervention is a therapeutic programme that consists of physical training and education and is led by experienced physiotherapists. It has been evaluated in previous studies for adverse effects and is considered low-risk and safe.

In the planned measurements, the mental stress of filling out the questionnaires can be described as low to moderate: you have to concentrate for 30-60 minutes. The physical stress from the performance tests can also be described as low to moderate. Physical exertion is not required. The performance of the performance tests takes about 20 to 30 minutes including breaks.

Possible benefits when participating in the study

The benefit to your health from your participation cannot be estimated at this time. However, the results of this study should help,

- that the treatment of future patients with osteoarthitis is improved by avoiding under- and inappropriate care,
- that health care expenditure is reduced and the burden on the insurance pool is eased,
- that the *"SmArt-E"* programme reduces unnecessary surgeries.

Data protection

Personal data will be collected from you at the measurement appointments and via the app. This data is pseudonymised at the University of Bremen in the trust centre of the KKSB, which means data by which you can be clearly identified, such as your name, is replaced by an identification number. In this way, your data cannot be traced back to you personally. In addition, selected health care data is transmitted by your health insurance fund to the trust centre of the KKSB. This data is also pseudonymised there.

All data collected from you will only be used for research purposes and the evaluated data will only be published in anonymous form. Your personal data will be deleted after completion of the study and your data set will be stored exclusively in anonymised form. Should you withdraw your consent to participate, your personal data will be deleted.

Contact details

If you have any questions about this study, please contact:

Name:	Franziska Weber	Carsten Müller
Telephone:	0234 / 777 27 -625	0234 / 777 27 -762
E-Mail:	smart-e@hs-gesundheit.de	

Informed consent form

SmArt-E: *Smartphone-assisted physical training with education*

Name in block letters: ....

- I have been informed by Mr/Ms _______________ about the nature, importance and scope of the study and the requirements arising for me from it. I have also read the text of the patient information leaflet and this declaration of informed consent.
- I had enough time to ask questions and make decisions. Questions that arose were answered by the study staff.
- I know that I can end my voluntary participation at any time without suffering any disadvantages.

I agree to participate in the study.

Data protection

I agree that for the purpose of the above study, my personal data, in particular information about my health, will be collected in the study centers or at the participating physiotherapy practices and via an app and transmitted to the KKSB trust office. This data may be passed on to the University of Bremen and the Ruhr University Bochum in pseudonymised (that means encrypted) form for scientific evaluation. The pseudonymised data will be stored for 10 years at the:

Competence Center for Clinical Trials Bremen (KKSB)

Department 03 I Mathematics and Computer Science

Linzer Strasse 4

28359 Bremen.

I agree that authorised persons who are subject to professional confidentiality (e.g. monitors of the client) may view my original data in order to verify the proper conduct of the study.

I have been informed that I can withdraw my consent at any time without giving reasons. In the event of a study withdrawal, I can decide whether my already collected data must be deleted or may be further used in anonymised form.
I have received a copy of the participant information and informed consent form. The original remains with the study staff.

________________________ Date and Signature participant

________________________ Date and Signature of study staff
